# PM10 Alters Trophoblast Cell Function and Modulates miR-125b-5p Expression

**DOI:** 10.1155/2022/3697944

**Published:** 2022-01-07

**Authors:** Wittaya Chaiwangyen, Komsak Pintha, Payungsak Tantipaiboonwong, Piyawan Nuntaboon, Orawan Khantamat, Francisco Lázaro Pereira de Sousa

**Affiliations:** ^1^Division of Biochemistry, School of Medical Sciences, University of Phayao, Phayao 56000, Thailand; ^2^Department of Biochemistry, Faculty of Medicine, Chiang Mai University, Chiang Mai 50200, Thailand; ^3^Department of Gynecology and Obstetrics, UNILUS (Centro Universitário Lusíada), Santos, Brazil 11050-071

## Abstract

Air pollution is one of the largest global environmental health hazards that threaten premature mortality or morbidity. Particulate matter 10 (PM10) has been demonstrated to contribute to several human diseases via dysregulated miRNA expression. Trophoblast cells play a key role in implantation and placentation for a successful pregnancy. Nonetheless, the PM10 associated trophoblast cell functions during pregnancy and miRNA expression are still unknown. Our study showed that PM10 affected HTR-8/SVneo cell viability and also decreased cell proliferation, migration, and invasion. A high concentration of PM10 caused an increase in HTR-8/SVneo cell apoptosis. Treatment with PM10 induced inflammation through the upregulated IL-1*β*, IL-6, and TNF-*α* expression in trophoblast cells. In PM10-treated HTR-8/SVneo cells, miR-125b-5p expression was considerably increased and TXNRD1 was found to be negatively related to miR-125b-5p. Collectively, our findings revealed that PM10 could alter miR-125b-5p expression by targeting TXNRD1 and suppressing trophoblast cell functions. Additional investigations relating to the function of miR-125b-5p and its target on particulate pollution exposure in trophoblast are warranted for future biomarker or effective therapeutic approaches.

## 1. Introduction

The top five health risks include air pollution with an elevated level of particulate matter (PM), which leads to premature death throughout the globe [1, 2]. PM is composed of airborne particles with a combination of liquid and solid particles including endotoxins, sulfates, nitrates, carbon, iron, zinc, sodium, potassium, ammonium, calcium, copper, lead, nickel, and polycyclic aromatic hydrocarbons (PAHs) [3, 4]. Generally, PM is classified by size into ultrafine particles with a diameter of less than 0.1 *μ*m (PM0.1 or UFPs), fine particles with a diameter less than 2.5 *μ*m (PM2.5), and coarse particles with a diameter less than 10 *μ*m (PM10), which are formed naturally through forest fire, sulfates from volcanoes, desert dust, and sea salt [2, 4, 5]. An increase in anthropogenic disturbances also induced PM levels such as combustion, industrial, vehicle exhaust emissions, agricultural activities, mining processes, and construction [2, 4].

Mostly, the primary effect of PM toxicity is related to oxidative stress, which contributes to transcription factor activation and further leads to local or systematic inflammation through the induction of proinflammatory responses such as nuclear factor kappa-light-chain-enhancer of activated B cells (NF-*κ*B), interleukin-1 (IL-1), IL-6, IL-8, cyclooxygenase-2 (COX-2), and granulocyte-macrophage colony-stimulating factor (GMCF) [2, 6–8]. Inhaled PM has the ability to enter terminal alveoli and blood circulation via the blood-air barrier, affecting the human respiratory system, central nervous system, reproductive systems, and cardiovascular system [9–16].

During implantation, extravillous trophoblast (EVT) cells invade the maternal decidua and myometrium for remodeling of maternal spiral arteries, leading to increased width of spiral arteries, nutrient supply, and gas exchange for fetal development [17, 18]. Several studies have shown that the impairment of trophoblast invasion is associated with preterm birth (PTB), preeclampsia (PE), spontaneous abortion, and fetal growth restriction (FGR) [19, 20].

MicroRNA (miRNA) is a class of short, single-stranded, noncoding RNA with 21-23 nucleotides that bind specifically to their target mRNA at 3′UTR for gene silencing through mRNA degradation or translational repression [21, 22]. miRNAs have been shown to be crucial for cellular functions and human development, including cell proliferation, migration, invasion, differentiation, angiogenesis, and apoptosis [23–25]. Accumulating evidence has revealed that aberrant expression of miRNAs is associated with diseases such as cardiovascular diseases, autoimmune diseases, inflammation, cancer, metabolic diseases, and pregnancy complications [26–28]. miR-125b-5p has been linked to hypoxia, oxidative stress, and inflammation [29]. In addition, miR-125b-5p was shown to be downregulated in pregnant women with FGR, PE, gestational diabetes mellitus (GDM), and gestational hypertension [30–32]. It has been shown that erythropoietin (Epo) was found to be targeted by miR-125b-5p, and decreased miR-125b-5p expression has been found in PE [33].

As a consequence of PM exposure, numerous organs, including the brain, kidney, heart, lung, and reproductive system, are potential targets of PM toxicity [34–36]. PM2.5 and PM10 showed a reduction in *β*-HCG secretion and cell growth, whereas they induced inflammation, endoplasmic reticulum stress, and oxidative stress in the trophoblast cell line [37]. This result was consistent with a previous study that found that PM2.5 exposure induced oxidative stress and inflammation and also decreased *β*-HCG secretion in trophoblast cells [38]. In addition, an increase in trophoblast cell cycle arrest and inhibition of cell invasion, and migration through downregulating Collagen I expression and upregulating TIMP1 and TIMP2 expression was observed upon PM2.5 treatments [39].

Up to date, several investigations have demonstrated that PM2.5 and PM10 alter miRNA expression profiling and are also involved in human pathology, including cardiovascular diseases, cancer, neurodegenerative diseases, and pulmonary diseases [40]. However, there was no evidence of PM10 on miRNA expression in trophoblast cells. We hypothesized that PM10 exposure may modulate trophoblast cell functions through altering trophoblast miRNA expression during an inflammatory response.

## 2. Materials and Methods

### 2.1. PM10 Collection

The samples of PM10 were collected at the University of Phayao, Phayao, Thailand, during the summer of 2019 with a high incidence of forest fire. The samples were collected on 20.3 × 25.4 cm quartz-fiber filters (Toyo Roshi Kaisha, Ltd., Tokyo, Japan) by using an Ecotech Model 3000 PM10 high volume air sampler with the flow rate set at 1.13 m^3^/min (67.8 m^3^/hour) for 24 h (Ecotech Pty. Ltd., Melbourne, Australia). The membranes were kept at -20°C until being used.

### 2.2. PM10 Extraction

The filter samples were cut into small pieces and extracted with a mixture of hexane and dichloromethane (1 : 1) for 15 min in an ultrasonic bath. Subsequently, the sample was filtered with a 0.45 *μ*m Polytetrafluoroethylene (PTFE) filter to eliminate unsolvable compounds. Then, the filtered sample was evaporated using a rotary evaporator and stored at -20°C until analysis [41, 42].

### 2.3. Particulate Matter Size and Element Analysis

The particle size and chemical composition were carried out using scanning electron microscopy (SEM) (FEI Quanta FEG 250, FEI Company, WA, USA) with an integrated energy-dispersive X-ray system (EDX) (Oxford INCA X-Act, Oxford Instruments, Buckinghamshire, UK) at the central laboratory, University of Phayao, Phayao, Thailand. For SEM analysis, 1 mm × 1 mm filter samples were cut from the middle of the filter. Using a gold sputter coater with a vacuum coating system (Quorum SC7620, East Sussex, UK), a gold (Au) thin film was applied to the sample surface. xT microscope control software was used to analyze the micrographs of particulate matter. For quantitative chemical composition of PM10, EDX spectra of PM10 were recorded and the weight percentage of each element existing in the spectrum was analyzed. Twelve elements were identified by SEM-EDX using Oxford Aztec software, including sodium (Na), carbon (C), nitrogen (N), oxygen (O), magnesium (Mg), silicon (Si), aluminum (Al), calcium (Ca), sulfur (S), iron (Fe), potassium (K), and chloride (Cl).

### 2.4. Cell Line

The trophoblast cell line HTR-8/SVneo was a kind gift from Prof. Charles H. Graham, Kingston, Canada, and was cultured in RPMI medium (Thermo Fisher Scientific, Dreieich, Germany). The medium was supplemented with 10% heat-activated fetal bovine serum (FBS; Sigma-Aldrich, Darmstadt, Germany) and 1% penicillin/streptomycin (Thermo Fisher Scientific, Dreieich, Germany) at 5% CO_2_, 37°C.

### 2.5. Cytotoxicity Assay

The cytotoxicity was performed using the 3-(4,5-dimethylthiazol-2-yl)-2,5-diphenyltetrazolium bromide (MTT) reduction assay (Sigma-Aldrich, Darmstadt, Germany). HTR-8/SVneo cells were seeded at a density of 1 × 10^4^ cells/well in a 96-well plate. Cells were exposed to varying concentrations of PM10 for 24-72 h, and a subsequent MTT solution (100 *μ*l) was added for another 4 h at 37°C. An equal amount of dimethyl sulfoxide (DMSO) was applied to dissolve the formazan crystals. The absorbance values were recorded at 570 nm using a microplate reader.

### 2.6. Matrigel Invasion and Transwell Migration Assay

Cell migration and invasion were analyzed with polyethylene terephthalate (PET) hanging cell culture inserts with 8 *μ*m pore size in a 24-well plate (Sigma-Aldrich, Darmstadt, Germany). For the invasion assay, HTR-8/SVneo cells (1 × 10^5^) were exposed to PM10 in serum-free RPMI-1640 medium and added to the Matrigel precoated membranes (Corning, AZ, USA) or transwell inserts for the migration assay. In the lower chamber of inserts, 20% FBS was supplemented to RPMI medium to be used as a chemoattractant. The invaded cells to the bottom membrane were fixed with 80% cold ethanol and stained with 0.1% *w*/*v* crystal violet. The remaining cells in the upper chamber were gradually detached using a cotton swab. After decolorizing the stained cells with 1% acetic acid, the absorbance was measured at 570 nm [43].

### 2.7. Proliferation Assay

A colorimetric BrdU incorporation ELISA kit (Sigma-Aldrich, Darmstadt, Germany) was performed to measure the proliferation assay. Briefly, HTR-8/SVneo cells were seeded in a 96-well plate at a density of 5 × 10^3^ cells/well and exposed to PM10 (5-50 *μ*g/ml) for 24-72 h. BrdU-containing medium was added for another 2 h. The BrdU-incorporated cells were fixed and incubated with a monoclonal anti-BrdU antibody conjugated with peroxidase. Following the washing steps, the cells were incubated with substrate before being stopped with 1 M H_2_SO_4_. Absorbance was measured at 450/690 nm using a microplate reader [44].

### 2.8. Apoptosis Assay

Apoptosis was performed using Alexa Fluor™ 488-annexin V and Propidium Iodide (PI) Dead Cell Apoptosis Kit (Life Technologies, Carlsbad, USA) following the manufacturer's guidelines. Cells were exposed to PM10 for 24 h and the subsequence resuspended at a density of 1 × 10^6^ cells/ml in 1x annexin-binding buffer. Cell suspensions were incubated with Alexa Fluor 488-annexin V and PI in the dark for 15 min. Apoptotic cells were then evaluated using flow cytometry (Attune NxT, Thermo Fisher Scientific, Waltham, MA, USA).

### 2.9. RNA Isolation

Total RNA was extracted from either PM10-treated or nontreated cells using the TRIzol reagent (Invitrogen, Darmstadt, Germany) following the manufacturer's protocols. Total RNA concentrations were monitored with a NanoDrop spectrophotometer (PeqLab Biotechnologies GmbH, Erlangen, Germany), and the A260/A280 ratio for all samples greater than 1.8 was stored at -80°C until being used [44].

### 2.10. Quantification of miRNA Expression by Quantitative RT-PCR (qRT-PCR)

cDNA synthesis was performed by using the miRCURY LNA Reverse Transcription Kit (Qiagen, Düsseldorf, Germany) following the manufacturer's protocol. Then, qRT-PCR was performed in QIAquant™ 96 (Qiagen, Düsseldorf, Germany) by using the miRCURY LNA SYBR Green PCR kit according to the manufacturer's protocol (Qiagen, Düsseldorf, Germany). All primers were purchased from Qiagen: miR-125b-5p (YP00205713) and RNU48 (YP0020/NR_002745) (Qiagen, Düsseldorf, Germany). Expression of miR-125b-5p was calculated using the 2^-*ΔΔ*Ct^ method, using RNU48 as a reference.

### 2.11. miRNA Target Prediction

Potential targets of miRNA were performed using a miRNA target prediction database including PicTar (https://pictar.mdc-berlin.de), miRDB (http://mirdb.org), and TargetScanHuman (http://www.targetscan.org/vert_71/).

### 2.12. Quantitative RT-PCR (qRT-PCR)

RNA was reverse-transcribed into cDNA using oligo-dT primers and RevertAid RT Reverse Transcription Kit (Thermo Fisher Scientific, Waltham, MA, USA), following the manufacturer's protocols. The expression of IL-1*β*, IL-6, TNF-*α*, DRAM2, TNFSF4, TRAF6, and TXNRD1 was evaluated with specific primers (listed in Table 1). qRT-PCR was performed using Maxima SYBR Green qPCR Master Mix (Thermo Fisher Scientific, Waltham, MA, USA) according to the manufacturer's protocols on QIAguant™ 96 (Qiagen, Hombrechtikon, Switzerland). Gene expression was quantified using the 2^-*ΔΔ*Ct^ method and normalized with GAPDH.

### 2.13. Statistical Analysis

The data was represented as the mean ± SD of three independent experiments. The data was analyzed by one-way analysis of variance (ANOVA), and a *P* value < 0.05 considered statistically significant was used for all statistical analyses using the GraphPad Prism version 8.0 (GraphPad Software, CA, USA).

## 3. Results

### 3.1. Size and Element Composition of PM10

**Figure 1:**
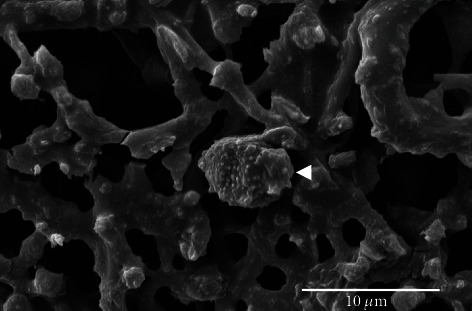
SEM image of PM10 (white arrow) at 12,000x magnification.

**Figure 2:**
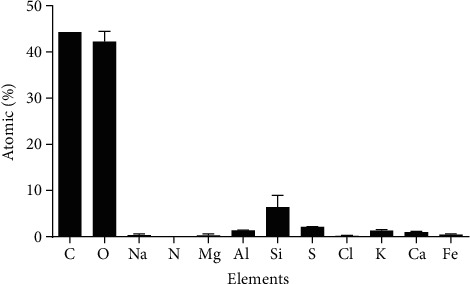
Element constituents in PM10 samples (atomic percentage) using SEM-EDX.

To analyze the size and element composition of PM10, samples of PM10 were cut into tiny pieces and identified using SEM-EDX. We found that the size distribution of PM10 ranged between 5.2 and 7.0 *μ*m and presented a near-spherical shape (Figure 1). Shown in Figure 2 is the distribution of atomic percent of C, O, Na, N, Mg, Al, Cl, Fe, Si, S, K, and Ca. The result showed that C (44.28%) and O (42.21%) were the main elements in PM10, followed by Si (6.38%) and other elements.

### 3.2. Effect of PM10 on Trophoblast Cell Viability

**Figure 3:**
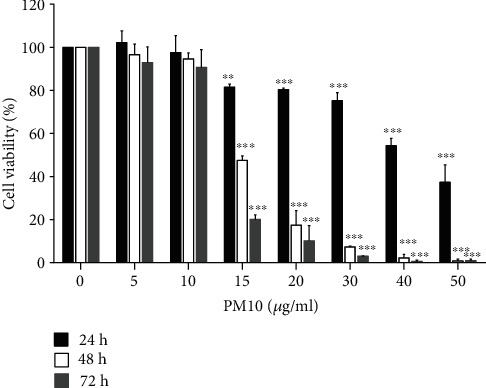
Cytotoxicity of PM10 on HTR-8/SVneo cells. Cells were exposed to PM10 (5-50 *μ*g/ml), and cell viability was evaluated at 24-72 h using the MTT assay.

HTR-8/SVneo cells were initially exposed to 5-50 *μ*g/ml of PM10 for 24-72 h, and the cytotoxic effect of PM10 was assessed using an MTT assay. Our results demonstrated that PM10 exposure significantly decreased HTR-8/SVneo cell viability in a dose-dependent manner at concentrations of 15-50 *μ*g/ml for 24-72 h (Figure 3). The nontoxic concentrations of PM10 (>80% cell viability) were selected for further study.

### 3.3. PM10 Attenuates Trophoblast Cell Proliferation, Migration, and Invasion

**Figure 4:**
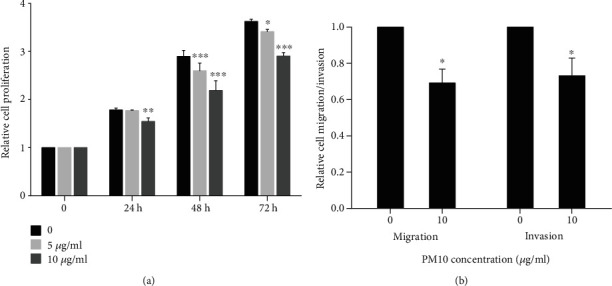
Effect of PM10 on HTR-8/SVneo cell proliferation, migration, and invasion. Cells were exposed to PM10 (5 and 10 *μ*g/ml) at 24-72 h for (a) proliferation assay and for (b) migration and invasion assays.

To further analyze whether PM10 can alter trophoblast cell functions, which are crucial steps for the implantation process, HTR-8/SVneo cells were exposed to PM10, and subsequent cell proliferation, migration, and invasion assays were performed. The results revealed that a low concentration of PM10 at 5 *μ*g/ml suppressed HTR-8/SVneo cell proliferation at 48-72 h, whereas a high concentration of PM10 exposure at 10 *μ*g/ml decreased cell proliferation at 24-72 h (Figure 4(a)). It appeared that PM10 exposure at 5 *μ*g/ml had no effect on trophoblast cell migration and invasion (results not shown), while 10 *μ*g/ml dramatically decreased HTR-8/SVneo cell migration and invasion (Figure 4(b)). Our findings showed that PM10 suppressed trophoblast cell proliferation, migration, and invasion.

### 3.4. Effect of PM10 on Trophoblast Cell Apoptosis

**Figure 5:**
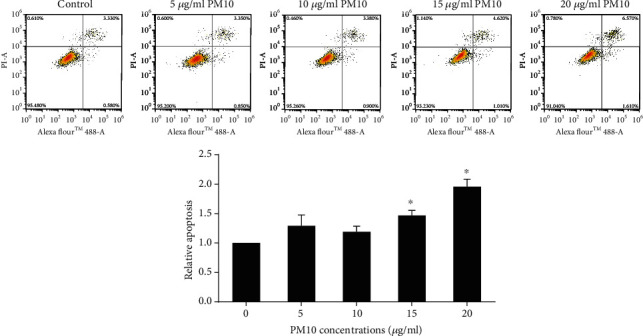
Effect of PM10 on HTR-8/SVneo cell apoptosis. Cells were exposed to PM10 for 24 h, and cell apoptosis was analyzed using flow cytometry.

The effect of PM10 exposure on trophoblast cell apoptosis was next investigated. Cell apoptosis was measured using flow cytometry upon cells being exposed to PM10. The nontoxic doses of PM10 had no influence on HTR-8/SVneo cell apoptosis (Figure 5). In comparison to a nontoxic dose, 15 and 20 *μ*g/ml of PM10-treated cells promoted HTR-8/SVneo cell apoptosis. These data suggest that nontoxic doses of PM10 were not associated with trophoblast cell apoptosis.

### 3.5. PM10 Exposure Induces Proinflammatory Cytokine Expression

**Figure 6:**
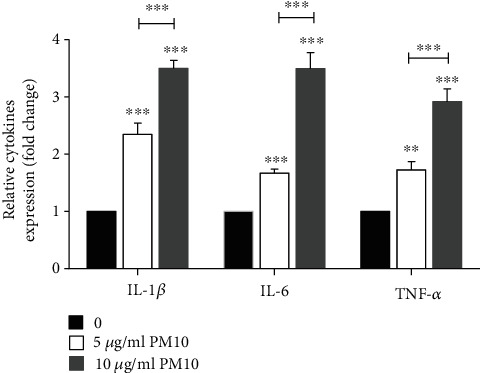
Effects of PM10 on proinflammatory cytokine expression in HTR-8/SVneo cells. Cells were exposed to PM10 for 24 h, and the expression of cytokines was performed using qRT-PCR.

It has been revealed that PMs could induce oxidative stress and inflammation through the expression of multiple cytokines [45, 46]. Therefore, we analyzed the expression levels of IL-1*β*, IL-6, and TNF-*α* in HTR-8/SVneo cells following 24 h of PM10 exposure. The qRT-PCR data showed that PM10 significantly induced IL-1*β*, IL-6, and TNF-*α* in a dose-dependent manner, as shown in Figure 6. All cytokines were markedly induced by 10 *μ*g/ml of PM10 when compared to 5 *μ*g/ml of PM10. These findings suggest that PM10 may be responsible for inflammation during pregnancy upon PM10 exposure.

### 3.6. PM10 Downregulates miR-125b-5p Expression and Targeting TXNRD1 in Trophoblast Cells

**Figure 7:**
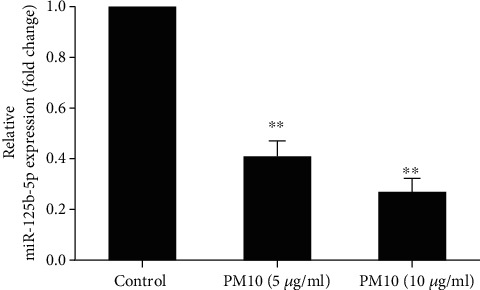
Effect of PM10 on miR-125b-5p expression in HTR-8/SVneo. Cells were incubated with 5-10 *μ*g/ml of PM10 for 24 h. The expression of miR-125b-5p was performed using qRT-PCR.

**Figure 8:**
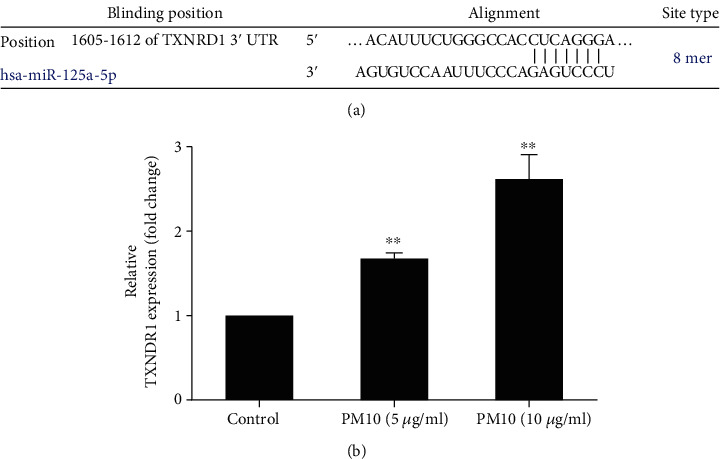
Potential target of miR-125b-5p in HTR-8/SVneo. (a) miR-125b-5p binding site with TXNRD1 predicted using the TargetScan database. (b) Cells were incubated with 5-10 *μ*g/ml of PM10 for 24 h. The expression of TXNRD1 was performed using qRT-PCR.

As miRNAs contribute to trophoblast cell functions, and to further verify that PM10 can alter miRNA expression, cells were exposed to PM10 for 24 h and the expression levels of miR-125b-5p were quantified using qRT-PCR. PM10 significantly suppressed miR-125b-5p expression (Figure 7). Subsequently, we further investigated the potential target of miR-125b-5p by using bioinformatics platforms including Pictar, miRDB, and TargetScan. According to TargetScan, miR-12b-5p targeted an 8 mer location in the 3′UTR of TXNRD1 at transcript positions 1605–1612 (Figure 8(a)). Therefore, TXNRD1 was selected as a potential target of miR-125b-5p, and TXNRD1 expression was confirmed by using RT-qPCR. There was an inverse relationship between TXNRD1 and miR-125b-5p expression in HTR-8/SVneo cells treated with PM10 at 5-10 *μ*g/ml (Figure 8(b)), implicating that TXNRD1 is one of the potential targets of miR-125b-5p in trophoblast cells. Other potential targets of miR-125b-5p associated with inflammation, DRAM2, TNFSF4, and TRAF6, were also investigated in this study. However, there were no significantly different expressions of these targets upon PM10 exposure in HTR-8/SVneo cells when compared to untreated cells (data not shown).

## 4. Discussion

Air pollution, especially particulate matter, has a significant influence on human health and the risk of developing respiratory diseases [47]. Besides, various diseases have also been associated with PM exposure, including cardiovascular diseases, cancer, neurological diseases, and pregnancy complications [48–51], leading to mortality and morbidity worldwide. There are 2 main routes for PMs to enter human circulation: (1) via the respiratory systems and (2) via the digestive tract [52]. Forest fires and burning seasons in Northern Thailand are generally seen from December to May. Between April and May is the peak of the fires, which cause air pollution and health problems [53–55]. The PM10 samples were collected during April-May in Phayao province, which is located in the north of Thailand. We found that the particle size was smaller than 10 *μ*m in diameter, indicating PM10. The main sources of PM10 in this study were forest fires and burning for traditional agriculture. These particles were mainly composed of C and O, and minor concentrations were Si, Na, Mg, S, Fe, Al, K, Cl, and Ca, which is consistent with a previous study showing that C and O were the major compositions of particles from Bangkok, Thailand [53]. However, the composition and concentration of elements in PM10 also depend on the combustion of different plants [56, 57].

Trophoblast cells are a unique cell lineage that invades into the maternal decidua for the establishment of fetomaternal circulation and also increases blood flow (maternal blood to adequately perfuse the placenta) adequately to supply the fetus [58]. Defects of trophoblast invasion are associated with pregnancy complications, including PE and IUGR [59]. Interestingly, it has been reported that black carbon particles that derive from air pollution translocated and accumulated on the fetal side of the placenta, indicating that direct exposure of black carbon particles to the fetus has a negative impact on fetal development [60]. Several studies found that exposure to PM10 was associated with PTB and low birthweight [61–64]. However, the biological mechanisms involved in the potentially mediating processes of air pollution exposure are largely unexplored. We hypothesized that PM10 exposure might be responsible for the alteration of inflammatory cytokine expression, trophoblast cell functions, and miRNA expression. In the present study, we found that PM10 had a cytotoxic concentration on first trimester trophoblast HTR-8/SVneo cells of more than 10 *μ*g/ml. Thus, 5-10 *μ*g/ml of PM10 was selected for further studies into the putative miRNA-mediated trophoblast cell functions associated with PM10 exposure. We discovered the effects of PM10 on trophoblast cell function in which our results revealed that PM10 dramatically suppressed HTR-8/SVneo cell proliferation, migration, and invasion. These results confirmed that PM10 exposure is capable of impairing trophoblast cell functions, and our data was consistent with previous studies that PM2.5/PM10 had a negative impact on trophoblast cell proliferation, migration, and invasion [37, 39].

PM has been shown to induce a significant inflammatory response upon phagocytosis of PM by macrophages and airway epithelial cells, contributing to oxidative stress and systemic inflammation via secreted proinflammatory mediators [48]. These inflammatory mediators, including IL-1*α*, IL-1*β*, IL-5, IL-6, IL-8, IL-17, IL-18, COX-2, IFN-*γ*, TNF-*α*, granulocyte/macrophage colony-stimulating factor (GM-CSF), macrophage inflammatory protein-1*α* (MIP-1*α*), MIP-1*β*, and MIP-3*α*, in *in vitro* and *in vivo* studies, depend on the constitution of PM [45, 65–67]. We found elevated levels of IL-1*β*, IL-6, and TNF-*α* in HTR-8/SVneo cells treated with PM10 at 5-10 *μ*g/ml. The increase in IL-6 level following 0.5-5 *μ*g/ml PM10 exposure in HTR-8/SVneo cells was similar to our findings. PM10 exposure at 500-5000 ng/ml also significantly suppressed *β*-hCG expression in HTR-8/SVneo cells. Furthermore, exposure to 50 ng/ml of PM2.5/PM10 for 7 days could modify the expression trafficking proteins, which are PE- and IUGR-related proteins, contributing to ER stress activation, cell growth inhibition, oxidative stress, and inflammation [37]. The high levels of proinflammatory cytokines, particularly IL-1, IL-6, IL-8, and TNF-*α*, are associated with pregnancy complications, including PE, through impaired endothelial cell functions [68, 69]. To our knowledge, PM exposure can trigger inflammation and may develop the risk of pregnancy complications.

Nowadays, an increasing number of miRNAs have been explored for their biological functions and potential biomarkers in human diseases due to the significance of miRNAs in human biological processes, and aberrant miRNA expression is associated with diseases [70]. Notably, exposure to the environment may alter epigenetics, including miRNA, resulting in the development of human pathological conditions in the future [71]. Several studies have shown that changes in specific miRNA expression profiles respond to numerous environmental pollutants, including organic pollutants, metals, radiation, cigarette smoke, pesticides, carcinogens, and air pollution [72]. Therefore, altered miRNA expression is likely to be a biomarker as a result of their sensitivity to environmental pollution exposure. For example, blood samples of 143 participants from 4 European countries were collected, and the results showed that PM2.5 personal exposure has significant let-7d-5p, miR-24-3p, miR-425-5p, miR-502-5p, miR-505-3p, miR-4454, and miR-4763-3p expression [73]. These circulating miRNAs have the potential to be novel biomarkers for air pollution exposure.

It is well documented that during pregnancy, it is regulated by differential miRNAs, particularly placental-specific miRNAs, the chromosome 19 miRNA cluster (C19MC), and the chromosome 14 miRNA cluster (C14MC) [74–76]. Defects in miRNA expression are related to impaired trophoblast cell apoptosis, proliferation, migration, invasion, vascularization, and metabolism, contributing to pregnancy complications such as PTB, PE, and IUGR [25, 43, 44, 76, 77]. Accumulating evidence suggests that exposure to environmental toxicants during pregnancy induces alterations in placental miRNA expression [78, 79]. Data from a cohort study on the miRNA expression profile in the placenta upon exposure to arsenic (As), bisphenol A (BPA), cadmium (Cd), dichlorodiphenyldichloroethylene (DDE), lead (Pb), mercury (Hg), polybrominated diphenyl ethers (PBDEs), and polychlorinated biphenyls (PCBs) has demonstrated the downregulation of let-7 family, miR-10a, miR-96, miR-151p, miR-190, miR-252a, miR-193b, miR-423-5p, miR-518d-5p, miR-520a-5p, miR-520d-3p, and miR-1975. In addition, exposure to Pb led to upregulated miR-651, while downregulating let-7 family, miR-10a, miR-190, miR-146a, and miR-431. PCB and Cd exposure was correlated with the expression of miR-1537. Surprisingly, miR-23a, miR-517a, miR-517c, and miR-522 are all members of the C19MC, which had the highest expression among 112 miRNAs studied in the placenta after environment pollution exposure [80].

Tsamou et al. reported that PM2.5 exposure upregulated miR-20a and miR-21 in the placenta during the first trimester pregnancy, whereas it downregulated miR-16, miR-21, miR-146a, and miR-222 in the placenta during the second trimester of pregnancy. Moreover, placental miR-146a was downregulated in the third trimester of pregnancy upon PM2.5 exposure [81]. Thus, it revealed that PM exposure could alter miRNA expression in pregnancy. Although there have been extensive studies of differential miRNA expression in various diseases, the mechanism of air pollution and PM-induced miRNA alteration in pregnancy is still largely unclear. Thus, a deep insight into miRNA regulatory mechanisms in the association of PM on trophoblast cell functions may be potentially useful for improving preventive or therapeutic strategies and biomarkers for pregnancy complications with regard to environmental pollutants. It has been revealed that miR-125b-5p plays a key role in regulating inflammatory responses by targeting the NF-*κ*B signaling pathway [82]. Moreover, dysregulated miR-125b-5p expression has been reported in several cancers such as bladder cancer, breast cancer, gastric cancer, and hepatocellular carcinoma by regulating cancer cell proliferation, cell cycle, cell apoptosis, differentiation, migration, and invasion [83, 84]. In pregnancy, miR-125b-5p was upregulated in the third trimester placenta compared to the first trimester placenta, especially that it was predominantly expressed in trophoblast cells [85]. Low expression of miR-125b-5p has been reported in pregnancy complications, such as FGR, PE, GDM, and gestational hypertension [30–32]. Recently, dysregulated miR-125b-5p expression has been shown to impair trophoblast invasion in PE, suggesting a biomarker for pregnancy complications [86]. To determine the relevance of miRNA in PM10-induced inflammatory responses, HTR-8/SVneo cells were evaluated for miR-125b-5p expression. We demonstrated that PM10 significantly suppressed the expression of miR-125b-5p compared to untreated trophoblast cells. Our data suggests that PM10 could modify gene expression via downregulating miR-125b-5p expression, contributing to the suppression of trophoblast cell functions.

We then analyzed a potential target of miR-125b-5p, which is related to inflammation. The results by using the miRNA target prediction database and validation with qRT-PCR confirmed that thioredoxin reductase 1 (TXNRD1) was a potential target of miR-125b-5p, and its expression was negatively regulated by miR-125b-5p. Our finding was consistent with a previous study that showed TXNRD1 was a target of miR-125b-5p [83]. TXNRD1 belongs to the pyrimidine nucleotide oxidoreductases family in the thioredoxin system and is a cytoplasmic antioxidant enzyme that regulates redox homeostasis [87, 88]. Overexpression of TNXRD1 is associated with highly proliferative cells and high ROS levels, such as cancer cells, contributing to protecting cells from ROS [89]. S-(1,2-Dichlorovinyl)-l-cysteine (DCVC) is an active metabolite of the environmental toxicant trichloroethylene (TCE), which increases ROS, IL-6, and TNXRD1 in HTR-8/SVneo-treated cells [90]. HTR-8/SVneo cells treated with the main flame retardant, 2,2′,4,4′-tetrabromodiphenyl ether (BDE-47) for 24 h, showed the upregulation of TXNRD1 [91]. It has been reported that high expression of TXNRD1 in the placenta was related to PE [92]. Our findings show that TXNRD1 overexpression in PM10-induced trophoblast cells is associated with inflammation, implying that TXNRD1 may result in limiting oxidative damage and preventing trophoblast cell apoptosis.

Taken together, PM10 exposure suppressed HTR-8/SVneo cell proliferation, migration, invasion, and dysregulated inflammatory responses, leading to impaired trophoblast functions, which are critical for placental development and successful pregnancy. We identified miRNA related to the inflammatory response of PM10-induced trophoblast, miR-125b-5p, resulting in altered TXNRD1 expression, which may enhance ROS elimination. Our findings suggest a possible role of particulate pollution on trophoblast functions and gene expression, which may help to understand the underlying mechanisms of air pollution in association with pregnancy complications. Further studies are required to identify additional signaling pathways and discover biomarkers and therapeutic or preventive strategies.

## Figures and Tables

**Table 1:** Sequences of specific primers.

Gene	Sequence 5′-3′
IL-1*β*	F: GCACAGTTCCCCAACTGGTA
	R: AAGACACGGGTTCCATGGTG
IL-6	F: AGACAGCCACTCACCTCTTCAG
	R: TTCTGCCAGTGCCTCTTTGCTG
TNF-*α*	F: CCCAGGCAGTCAGATCATCTTC
	R: AGCTGCCCCTCAGCTTGA
DRAM2	F: CCTTTCCTACCAAATGCAGCCC
	R: GCCACTGTGCAAAACTGATGAGC
TNFSF4	F: GTGCACCGGAGTTCTGTGT
	R: TTGCAGGGTAGTCGATGAC
TRAF6	F: TCATTATGATCTGGACTGCCCAAC
	R: TGCAAGTGTCGTGCCAAGTG
TXNRD1	F: AGGGCAGACTTCAAAAGCTACTAA
	R: ATATTGGGCTGCCTCCTTAGC
GAPDH	F: AGCCACATCGCTCAGACAC
	R: GCCCAATACGACCAAATCC

## Data Availability

The data that support the findings of this study are available on request from the corresponding author.
